# High Occurrence of Bacterial Competition Among Clinically Documented Opportunistic Pathogens Including *Achromobacter xylosoxidans* in Cystic Fibrosis

**DOI:** 10.3389/fmicb.2020.558160

**Published:** 2020-09-10

**Authors:** Quentin Menetrey, Chloé Dupont, Raphaël Chiron, Estelle Jumas-Bilak, Hélène Marchandin

**Affiliations:** ^1^HydroSciences Montpellier, Univ Montpellier, CNRS, IRD, Montpellier, France; ^2^HydroSciences Montpellier, Univ Montpellier, CNRS, IRD, Laboratoire d’Ecologie Microbienne Hospitalière, CHU Montpellier, Montpellier, France; ^3^HydroSciences Montpellier, Univ Montpellier, CNRS, IRD, Centre de Ressources et de Compétences de la Mucoviscidose, CHU Montpellier, Montpellier, France; ^4^HydroSciences Montpellier, Univ Montpellier, CNRS, IRD, Département de Microbiologie, CHU Nîmes, Nîmes, France

**Keywords:** cystic fibrosis, opportunistic pathogens, *Achromobacter*, *Stenotrophomonas*, competition, growth, motility, biofilm

## Abstract

Cystic Fibrosis (CF) airways favor abnormal microbial development. Infections are considered as polymicrobial and competition can be observed between microorganisms. The current literature on bacterial competition in CF mostly consists of studies with limited numbers of strains, mainly focused on the major pathogens *Pseudomonas aeruginosa* (*Pa*) and *Staphylococcus aureus* (*Sa*) and does not give a comprehensive overview of the overall importance of bacterial interactions or the behavior of less often encountered emerging bacteria such as *Achromobacter*. In this context, we screened a panel of 39 strains from six CF patients, of either clinical or domestic environmental origin, distinguished according to genotype and belonging to four opportunistic pathogens, *Pa* (*n* = 15), *Sa* (*n* = 3), *Stenotrophomonas maltophilia* (*Sm*, *n* = 10) and *Achromobacter xylosoxidans* (*Ax*, *n* = 11). We investigated their capacity to compete in terms of growth, motility, and pigment production on agar media through 203 crossing experiments. Eleven strains selected via the initial screening results were further studied for competitive growth in liquid medium and biofilm formation. Competition was noted for 33% (67/203) of the pairs of strains with 85 modifications observed between monocultures and co-cultures, impacting growth (23.6%), motility (13.8%), and/or pigment production (6.1%). Under all conditions of the study (clinical, environmental strains; intra-, inter-patients; intra-, inter-species levels), competition was significantly more frequent among pairs of strains with at least one clinical strain. While *Pa* mainly outcompeted other species, in one patient with chronic colonization by *Ax* and sporadic colonization by *Pa*, we showed that some *Ax* inhibited the growth and pigmentation of *Pa* whereas biofilm formation was drastically reduced. Enlarging the panel of strains tested in competition assays gave new perspectives on the complex interactions taking place among the CF airway community. Indeed, the frequent occurrence of varied, strain-dependent interactions is revealed here. We report the first results of competition assays for *Ax* with the ability of certain strains to outcompete *Pa*. Our results are linked to the patient’s colonization history and question the importance of bacterial competitiveness in the colonization pattern of CF airways.

## Introduction

Cystic fibrosis (CF) is a genetic disease caused by a mutation in the Cystic Fibrosis Transmembrane conductance Regulator gene coding for a transmembrane channel allowing the transport of chloride ions. Different organs are affected, including the respiratory tract, where thickening mucus, mucociliary clearance defects, and a decrease in anti-microbial defenses are observed. These alterations allow a variety of microorganisms, of endogenous and environmental origin, to multiply in the cystic fibrosis respiratory tract (CFRT; Burns et al., 1998; Lyczak et al., 2002; Ciofu et al., 2013). The main opportunistic pathogens identified in young children with CF are *Staphylococcus aureus* and *Haemophilus influenzae* whereas *Pseudomonas aeruginosa* predominates in older patients (Registre français de la mucoviscidose – Bilan des données, 2017). In addition, certain other opportunistic bacterial pathogens of environmental origin are encountered in CF patients and, despite being less frequently identified, some of them, such as *Achromobacter xylosoxidans* and *Stenotrophomonas maltophilia* are considered as emerging (Lyczak et al., 2002; Hansen et al., 2009; Lambiase et al., 2011; Parkins and Floto, 2015; Esposito et al., 2017; Registre français de la mucoviscidose – Bilan des données, 2017). Cultivation-independent studies have led to increasing knowledge about the diversity of the bacterial community in CF lungs and its dynamics throughout the evolution of this disease. They have revealed a higher phylogenetic diversity than expected from cultivation-based studies, a diversity that progressively decreases despite the overall abundance of microbiota remaining high, and the existence of co-occurring bacterial networks suggestive of functional associations within the CF polymicrobial consortium (Filkins et al., 2015; Quinn et al., 2016). Pulmonary infections in CF patients have been considered as polymicrobial for more than a decade now (Sibley et al., 2006; Peters et al., 2012; O’Brien and Welch, 2019) and pulmonary exacerbations have been shown to be associated with modifications in the CF community and a disruption/fragmentation of bacterial networks observed during stable clinical status (Quinn et al., 2016). These microbiota-disease associations must also be interpreted in an ecological perspective as they take place in the pulmonary environment, i.e., a restricted space subjected to diverse selective pressures (immune system, acidic environment, antibiotic cures, etc.; Rogers et al., 2013).

Due to both the diversity of bacterial species and the abundance of the community present in CF airways, competition between species for space and nutrients is established through physical and chemical interactions (Høiby et al., 2010; Armbruster et al., 2019). These interactions can be mutualistic or synergistic as they can improve the coexistence of microorganisms on epithelial surfaces and also enable them to use metabolic by-products more efficiently (Whiley et al., 2014, 2015). Conversely, other microorganisms can develop antagonistic interactions, for example by secreting effectors that inhibit the growth of co-colonizing species (Bragonzi et al., 2012; Michelsen et al., 2014; Tognon et al., 2019). These interactions are facilitated within multi-species biofilms and involve intercellular communication via quorum sensing (QS) molecules able to modulate the expression of certain virulence factors, bacterial growth, and/or regulate the host’s immune system (Duan et al., 2003; Hogan et al., 2004; Williams and Cámara, 2009; Tashiro et al., 2013; Pollitt et al., 2014; Beaume et al., 2015; Whiley et al., 2015; O’Brien and Fothergill, 2017). However, despite an increasing interest in characterizing interactions between species, their clinical relevance and potential implications in the progression of polymicrobial pathologies are still poorly understood (He and Shi, 2009; Limoli and Hoffman, 2019).

In CF, it is likely that pathogens develop specific competitive strategies as they develop in a narrow, hostile environment subjected to many selection pressures (Schwab et al., 2014). Competition between microorganisms should thus to be considered as a dynamic phenomenon, highly dependent on the strains’ biotic and abiotic environment and the colonization history of the patient’s airways (Palmer et al., 2005; Andersen et al., 2015). A better knowledge and characterization of these bacterial interactions could help us to understand the dynamics of bacterial colonization of the respiratory tract in CF patients and therefore studies on clinically documented CF strains warrant more consideration. Indeed, most studies on the bacterial competition that may occur in the CF lung have focused on the two major CF pathogens, *P. aeruginosa* and *S. aureus* (Baldan et al., 2014; Filkins et al., 2015; Hotterbeekx et al., 2017; Limoli et al., 2017; Tognon et al., 2017) and available data have mostly been obtained from a limited number of strains, including reference strains that may not reflect the true behavior of clinical strains. In this context, the main objective of our study was to investigate the existence of interactions between clinically documented representatives of opportunistic pathogenic species colonizing the respiratory tract of CF patients and the domestic environment of CF patients, a potential source for airway colonization (Heirali et al., 2016), and to specify the importance and nature of these interactions. We studied important features that may be modified through bacterial competition to confer an advantage to one of the partners, i.e., growth, motility, biofilm production, and production of pigments as an indirect marker for *P. aeruginosa* virulence. A focus was then made on the competitive ability of *A. xylosoxidans*, which had never been studied before.

## Materials and Methods

### Biological Materials, Bacterial Strains, and Patients

The strains under study were isolated during routinely performed analyses of sputum samples from six CF patients (Patients A–F among whom Patients A, C, and F were three patients chronically colonized by *A. xylosoxidans* included from previous studies (Dupont et al., 2015, 2016, 2018) in which they were known as Patients 12, 2, and 5, respectively) attending the CF center at Montpellier University Hospital, France (*n* = 24) or from the domestic environment of three of these patients (*n* = 15; Table 1). Strains were stored frozen at −80°C. The 39 bacterial strains belonged to four species: 11 *A. xylosoxidans* (*Ax*) including one environmental strain; 15 non-mucoid *P. aeruginosa* (*Pa*) including seven environmental strains; 10 *S. maltophilia* (*Sm*) including seven environmental strains and three methicillin-susceptible *S. aureus* (*Sa*; Table 1). Bacterial strains were genotyped by multi locus sequence typing (MLST) as previously described (Enright et al., 2000; Curran et al., 2004; Kaiser et al., 2009; Spilker et al., 2012). Microbiological data resulting from patient follow-up were retrospectively recorded to specify the patient’s colonization profile (co-isolated species, colonization type). Chronic and intermittent colonization’s were defined according to the modified Leeds criteria by isolating bacterium from > 50% or ≤ 50% of respiratory samples collected over the previous 12 months for patients with at least four samples analyzed during that period, respectively (Lee et al., 2003). Sporadic colonization refers to a general definition of bacterial identification occurring at irregular scattered or isolated intervals.

**TABLE 1:** Isolation and genotypic characteristics of the 39 strains under study.

Patient	Strain^a^	Sampling Date (dd/mm/yyyy)	Origin and type of airway colonization^b^	Sequence type^c^	Co-isolated Species
**Patient A**	***Ax* 198**	11/04/2012	clin: chronic	327	*Pa*
	***Ax* 199**	‘’	clin: chronic	327	*Pa*
	***Ax* 200**	‘’	clin: chronic	327	*Pa*
	***Pa* I.14**	29/07/2010	clin: sporadic	244	*Sa*; *Ax*
	***Pa* I.31**	29/10/2010	clin: sporadic	27	*Sa*; *Ax*
	***Pa* II.17**	11/04/2012	clin: sporadic	1092	*Ax*; *Pa*
	***Pa* 50**	03/12/2015	env: bathroom sink water	27	
	*Pa* 51	‘’	env: bucket in the shower	27	
	*Pa* 57	‘’	env: shower plughole grid	27	
	*Pa* 69	‘’	env: first flush of tap water	27	
	*Sm* 49	‘’	env: shower plughole water	94	
	*Sm* 50	‘’	env: sink water, bathroom	334	
	*Sm* 57	‘’	env: shower plughole grid	334	
	*Sm* 69	‘’	env: first flush of tap water	94	
**Patient B**	*Pa* II.79	09/10/2013	clin: chronic	395	*Escherichia coli*; *Aspergillus* sp.
	*Sm* 8	11/06/2013	clin: sporadic	4	*Enterobacter cloacae*; *Sa*; *Aspergillus* sp., *Candida* sp.
**Patient C**	*Ax* 67	22/04/2014	clin: chronic	328	
	*Ax* 68	‘’	clin: chronic	328	
	*Ax* 69	‘’	clin: chronic	328	
	*Pa* IV.7	03/08/2015	clin: sporadic	160	
	*Pa* 19	06/01/2016	env: bathroom sink water	309	
	*Pa* 21	‘’	env: shower plughole water	606	
	*Sm* 19	‘’	env: bathroom sink water	300	
**Patient D**	*Pa* I.72	22/11/2011	clin: sporadic	1062	*Pa*; *Sa*; *Aspergillus fumigatus*
	***Pa* I.73**	‘’	clin: sporadic	1062	*Pa*; *Sa*; *A. fumigatus*
	*Sm* 29	22/10/2013	chronic	246	*Sa*; *A. fumigatus*; *Cupriavidus respiraculi*
	***Sa* 12**	22/11/2011	chronic	5	*Pa*; *Sa*; *A. fumigatus*
**Patient E**	***Pa* II.39**	16/10/2012	clin: sporadic	253	*Sm*; *Sa*; *A. fumigatus*
	***Sm* 18**	17/09/2013	clin: intermittent	5	*A. fumigatus*
	*Sa* 27	11/12/2012	clin: chronic	2199	
	*Sa* 52	03/12/2013	clin: chronic	34	
**Patient F**	*Ax* VII.40	18/11/2015	clin: chronic	27	*Citrobacter freundii*
	*Ax* VII.41	‘’	clin: chronic	27	*C. freundii*
	*Ax* VII.42	‘’	clin: chronic	27	*C. freundii*
	*Ax* VII.43	‘’	clin: chronic	27	*C. freundii*
	*Ax* 50	09/12/2015	env: bathroom sink water	175	
	*Sm* 57.1	‘’	env: washing machine water	486	
	*Sm* 57.2	‘’	env: washing machine water	486	
	*Pa* 57	‘’	env: washing machine water	252	

*^*a*^*Ax*, *Achromobacter xylosoxidans*; *Pa*, *Pseudomonas aeruginosa*; *Sa*, *Staphylococcus aureus*; *Sm*, *Stenotrophomonas maltophilia*. Strains are presented in the same order as Supplementary Table 1, in order of origin (clinical or environmental), then species (*Ax*, *Pa*, *Sm*, and *Sa*). No mucoid or small colony variants were included in this study. All *Sa* were methicillin-susceptible *Sa*. Strains in bold were selected for the in-depth study. ^*b*^clin, clinical isolate; env, environmental strain from the patient’s domestic environment (Dupont et al., 2018). The type of patient airway colonization is given for clinical isolates. ^*c*^ST, Sequence Type as determined by Multi Locus Sequence Typing. ST 486 of *Sm* is a previously undescribed ST that we deposited in the PubMLST database.*

### Study Design

**FIGURE 1:**
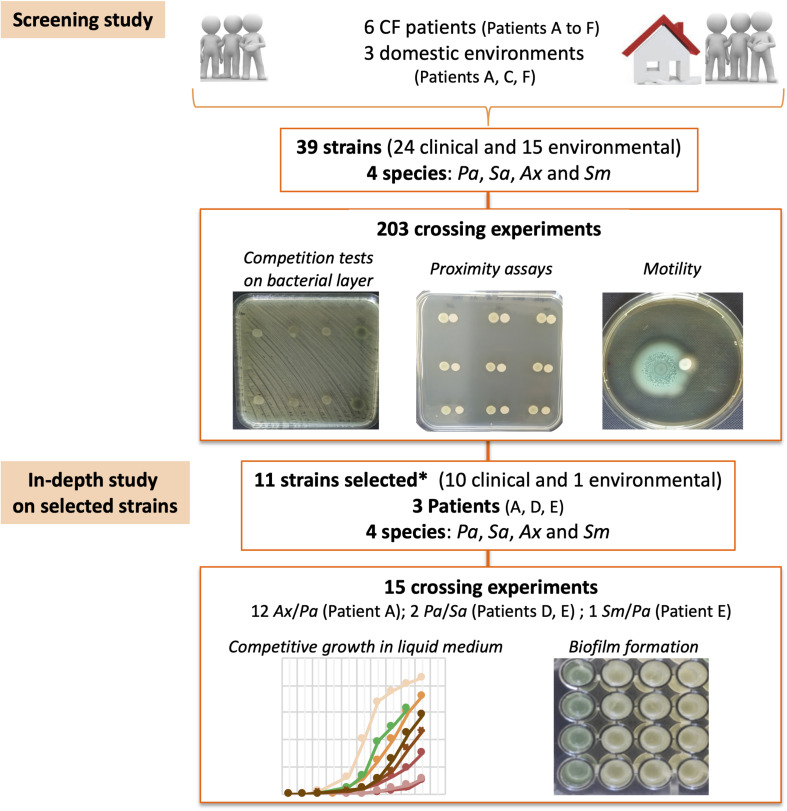
Study design flowchart. CF, Cystic Fibrosis; *Pa*, *Pseudomonas aeruginosa*; *Sa*, *Staphylococcus aureus*; *Ax*, *Achromobacter xylosoxidans*; *Sm*, *Stenotrophomonas maltophilia*. ^∗^ Selected strains were those for which the most important growth inhibition of one partner was observed during the screening experiments (two co-isolated strains were also included to investigate intra-sample diversity of *Ax*/*Pa* competition in Patient A).

Interactions between the 39 strains in our study were investigated through 203 crossing experiments to evaluate competition in terms of growth, motility, and global pigment production on agar media (Figure 1 and Supplementary Table 1). During this first part of the study (see “screening study” hereafter), all pairs of strains were tested by the three approaches detailed below: proximity assay, test on bacterial layer, and swimming motility evaluation. For assays including at least one *Pa* strain, these three assays simultaneously allowed us to visually detect any modification in pigment production by *Pa*. Among the 203 pairs of strains tested, 80.8% (164/203) included at least an emerging pathogen (*Ax* or *Sm*); 33 pairs included strains from the same species and 170 strains from different species, 121 came from the same patient and 82 from different patients, and 122 included strains of the same origin (clinical or environmental) while 81 included strains of different origins (clinical and environmental). All results obtained from the co-culture assays were compared with those obtained from monoculture assays on the two strains of the tested pair. Each experiment was performed at least twice. Based on the results obtained from this screening stage of our study, an in-depth study was then conducted on 11 selected strains originating from three patients to assess competition in terms of bacterial growth by establishing curves for growth and/or the ability to form biofilm, with each assay repeated 4 times (Figure 1 and Supplementary Table 1).

### Screening Study: Culture Conditions and Competition Assays

#### Culture Conditions

Bacteria were cultured on trypticase soy (TS) agar for 24 h at 37°C. Liquid cultures were then performed in TS broth incubated overnight at 37°C and shaken (175 rpm) under aerobic conditions (slightly open tubes). Before any experiment, the OD_600nm_ of each bacterial suspension was adjusted to 0.5, equivalent to 2–6.10^8^ colony forming units (CFU)/mL. Growth curves were established for the four species included in the study (*n* = 18 strains; 7 *Pa*, 1 *Sa*, 5 *Ax*, and 5 *Sm*). All the five *Ax* tested displayed a longer time to enter the exponential growth phase compared to other species (7 *Pa*, 1 *Sa*, and 5 *Sm*; Supplementary Figure 1), supporting the application of specific co-culture conditions for *Pa*–*Ax* pair testing, as in previous studies among species displaying distinct growth rates (Duan et al., 2003; Pompilio et al., 2015). Consequently, a time offset of 4 h was retained for *Ax* deposited 4 h before *Pa* in the various experiments, and the OD_600nm_ of *Pa* was adjusted to 0.1 to reduce the nutrient competition with *Ax*. Incubation was carried out at 37°C for 48 h for all assays. *Sm* strains showed heterogeneous growth characteristics and specific conditions were not applied to co-cultures including an *Sm* strain in this study (Supplementary Figure 1).

#### Proximity Assays

Tests were performed according to Whiley et al. (2015) with modifications: 10 μL-spots of two strains were deposited opposite each other, at three different distances, 0.4, 0.6, and 0.8 cm, respectively. Growth zones were visually observed to search for a decrease or potentiation of growth of the bacteria under study. Controls consisted of depositing each of the two strains in spots opposite each other.

#### Competition Tests on Bacterial Layer

Tests were performed according to Beaume et al. (2015) with modifications. Briefly, TS agar was inoculated by swabbing a bacterial suspension. Spots (10 μL) of a “competitor” bacterium in suspension were then deposited on the surface of the seeded agar. The diameter of the growth inhibition zones of the bacterial layer around the competitor bacterium’s spot was measured (in mm) and any modification to the spot’s aspect (growth, pigmentation) was recorded.

#### Motility

Swimming motility was studied according to Kirov et al. (2004) with modifications. Briefly, 10 μL of bacterial suspension was deposited on the appropriate semi-solid agar and the same volume of competitor strain in suspension was deposited 1.5 cm away from the first spot. Aspect and size of the competing bacteria’s motility zones were compared to those observed for the corresponding strain deposited alone on the agar surface and opposite itself.

### In-Depth Study on Selected Strains

This part of the study included crossing experiments conducted on 11 strains selected from three patients: nine strains belong to pairs of strains for which the most important growth inhibition of one partner was observed during the screening experiments; the two remaining strains, *Ax* 198 and *Ax* 199, were co-isolated with a selected strain and were included to complete the panel of strains in Patient A and to investigate intra-sample diversity of *Ax*/*Pa* competition (Table 1 and Supplementary Tables 1, 2). Strains were further studied for competitive growth in liquid medium and their ability to form biofilm. Pairwise comparisons were conducted for (i) clinical strains *Pa* II.39 and *Sm* 18 isolated about 1-year apart from Patient E, (ii) clinical strains *Pa* I.73 and *Sa* 12 isolated from the same sputum sample in Patient D, (iii) clinical strains *Pa* II.39 (Patient E) and *Sa* 12 (Patient D), and (iv) seven strains related to Patient A, i.e., three *Ax* clinical strains (*Ax* 198, *Ax* 199, *Ax* 200 corresponding to adaptive variants of identical MLST genotype but distinct colony morphotypes co-cultured from a sputum specimen in a chronically colonized patient), and 4 *Pa* (three clinical strains *Pa* I.14, *Pa* I.31, and *Pa* II.17, sporadically colonizing the patient’s airways, and one environmental strain, *Pa* 50). *Ax* 198, 199, and 200 were co-cultured from a sputum specimen sampled in April 2012 together with *Pa* strain II.17. Clinical strains *Pa* I.14 and *Pa* I.31 were cultured from sputum samples 2 years before, in July and October 2010, respectively. *Pa* 50 was recovered from water collected from the washbasin in the bathroom at Patient 12’s house in December 2015 (Supplementary Figure 2).

#### Competitive Growth in Liquid Medium

Overnight cultures were made to obtain strains at the beginning of the stationary phase as described in 2.4.1. OD_600nm_ of bacterial suspensions were adjusted to 0.5 (± 0.02) and each suspension was put into a 50 mL tube containing TS broth (1% of the total volume, 300 μL in 30 mL) with the following exception: for *Ax*/*Pa* co-culture experiments (and comparative *Pa* monoculture), the OD_600nm_ of *Pa* suspensions were adjusted to 0.1 (± 0.01) and the *Ax* bacterial suspension was inoculated in TS broth 4 h before the introduction of an equal volume of *Pa* suspension. The tubes were incubated at 37°C under aerobic conditions (slightly open tubes) and spun at 175 rpm for the duration of the experiment. Bacterial cells were enumerated (CFU/mL) after 24 and 48 h on TS agar (total count) and selective media (co-cultures) using EasySpiral Pro according to the manufacturer’s instructions (Interscience^®^). Based on the respective antimicrobial susceptibility profiles of the 11 selected strains, TS agar plates with either 5 mg/L of gentamicin or 8 mg/L of colistin were used as the selective media for *Ax*/*Sm* and *Sa*, respectively, after checking that similar growth was obtained on selective and non-selective media. *Pa* cells were enumerated on non-selective TS agar plates. To test the effect of culture supernatants, overnight cultures were centrifuged (10 min at 4000 rpm) then filtered using a 0.22 μm sterile supernatant filter. Fifteen mL of the supernatant were mixed with 15 ml of double strength TS broth before introducing the bacterial suspension to be tested. The conditions of further analysis were similar to those described above.

#### Biofilm Formation

Briefly, an overnight culture was grown aerobically in TS broth under agitation (175 rpm) at 37°C, then adjusted with sterile broth medium to an OD_600nm_ = 0.5 or to an OD_600nm_ = 0.1 for *Pa* when tested in *Pa*/*Ax* co-culture (and comparative *Pa* monoculture assay). Suspensions were diluted 100-fold. For dual-species cultures, these suspensions were mixed in a 1:1 ratio. Each well of a polystyrene 96-well microtiter plate (Nunc^®^) was seeded with 100 μL of standardized bacterial inoculum (single or dual cultures; Pompilio et al., 2015; Magalhães et al., 2017) and incubated for 48 h at 37°C, under aerobic conditions in a standard incubator. Biofilm quantification was performed according to Harvey et al. (2007) with modifications. Absorbance at 570 nm was determined prior to discarding the liquid content of the microtiter plate. The wells were then washed three times with tap water before adding crystal violet (CV) solution (1%). After washing, the CV was solubilized with 200 μL of ethanol 95° and 125 μL were transferred to a new microtiter plate for a new absorbance measure at 570 nm. Strains were categorized as non-adherent, weakly, moderately, or strongly adherent according to Stepanovic et al. (2000).

### Statistical Analysis

A Pearson’s chi-squared test was carried out for the comparison of results of the screening assays depending on the species and the condition, i.e., intra-patient versus inter-patient, intra-species versus inter-species, and origin of the strains (co-culture of clinical strains, co-culture of environmental strains, co-culture of clinical versus environmental strains). To compare bacterial concentrations observed in broth medium during co-cultures versus monocultures and values obtained during the evaluation of biofilm formation, the Friedman test (non-parametric ANOVA with paired samples) followed by a Dunn’s post-test was performed when at least three groups were compared. Otherwise, when both data groups followed a Gaussian distribution, a paired *t*-test was used as a parametric test. A Wilcoxon signed-rank test was used as a non-parametric test when at least one of the two groups did not follow a normal distribution. Data were analyzed using GraphPad Prism (GraphPad Software, La Jolla, CA). A two-tailed *P*-value < 0.05 was considered statistically significant (^∗^: *P*-value < 0.05, ^∗∗^: *P*-value < 0.01, ^∗∗∗^: *P*-value < 0.001).

## Results

### Bacterial Strains and Patients’ Bacterial Colonization Profiles

The 39 strains studied included four species isolated from 21 samples (13 clinical and eight environmental samples) belonging to 25 genotypes (two to five according to the species with multiple strains; Table 1). The panel of strains included various species co-isolated from one sample and up to three strains belonging to one species and originating from the same sample, either of different genotypes or of identical genotype, i.e., cultural variants. The six patients were chronically colonized by either one pathogen (patients B, C, E, and F) or two (patients A and D) and five of them had sporadic or intermittent colonization by other species (Table 1).

### Screening Study

#### Overall Results

A total of 85 modifications in growth, motility or pigment production were observed among the monoculture and co-culture tests for 67 of the 203 pairs of strains studied (33%). One modification was observed for 51 of the 67 pairs of strains (76.1%), two modifications for 14 of the 67 pairs (20.9%), and three modifications for two of the 57 pairs which had at least one *Pa* (3.5%).

Modifications in bacterial growth revealed either by the competition tests on bacterial layers or by the proximity assays were mostly observed (23.6% of the 203 crossing experiments) before changes in swimming motility (13.8%; Table 2). Induction or inhibition of overall pigment production by *Pa* strains was observed for 6.1% of pairs of strains that had at least one *Pa* (9/148), a notable decrease in pigmentation or even a complete absence of pigmentation being observed in seven of these cases. These modifications in *Pa* pigmentation were systematically associated with at least one other modification.

**TABLE 2:** Summary of the prevalence and type of interactions observed according to the crossing experiments.

Pairs of Species^a^	Pairs of Strains Tested (*n*)	Pairs of Strains Showing ≥ 1 Modification (*n*,%)	Pairs With Modifications Observed in Co-cultures (*n*, description^b^)		
			
			Growth	Motility	*Pa* pigment production
**Inter-species**					
*Pa*–*Ax*	53	26 (49%)	20 (*Pa* 9➚, 11➘)	11 (*Pa* 8➚, *Pa* 2➘, *Ax* 1➘)	7 (1 ➚, 6➘)
*Pa*–*Sm*	59	18 (31%)	14 (*Sm* 14➘)	7 (*Pa* 7➚)	2 (1➚, 1➘)
*Pa*–*Sa*	15	4 (26.7%)	4 (*Sa* 4➘)	0	0
*Ax*–*Sm*	36	5 (13.9%)	1 (*Sm* 1➘)	4 (*Sm* 4➘)	NA
*Sm*–*Sa*	7	1 (14.3%)	0	1 (*Sm* ➘)	NA

**Sub-total**	**170**	**54 (31.7%)**	**39**	**23**	**9**

**Intra-species**					
*Pa*–*Pa*	21	9 (42.8%)	9 (9➘)	1 (1➚)	0
*Ax*–*Ax*	6	4 (66.6%)	0	4 (4➚)	NA
*Sm*–*Sm*	3	0	–	–	NA
*Sa*–*Sa*	3	0	–	–	NA

**Sub-total**	**33**	**13 (39.3%)**	**9**	**5**	**0**

**Total**	**203**	**67**	**48**	**28**	**9**

*^*a*^*Ax*, *Achromobacter xylosoxidans*; *Pa*, *Pseudomonas aeruginosa*; *Sa*, *Staphylococcus aureus*; *Sm*, *Stenotrophomonas maltophilia*; NA, not applicable. ^*b*^➚, increase in the characteristic considered during competition assay; ➘, decrease in the characteristic considered during competition assay.*

**FIGURE 2:**
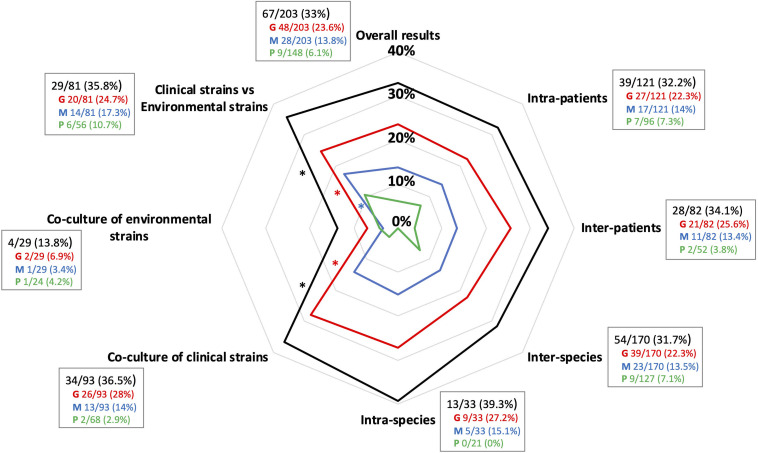
Rates of crossing experiments (%) showing competitive effects on growth, motility and *Pseudomonas aeruginosa* overall pigment production according to seven crossing conditions. Black, rate of pairs of strains showing at least one type of interaction; red, rate of pairs of strains showing interactions affecting growth; blue, rate of pairs of strains showing interactions affecting motility; green, rate of pairs of strains showing interactions affecting *P. aeruginosa* pigment production. Detailed results (number of pairs of strains showing an interaction out of the total crossing experiments performed in the category, %) are given next to the corresponding crossing condition with the same color code than above (G, growth; M, motility; P, *Pa* global pigment production). ^∗^, significant *P*-value < 0.05.

Interactions observed were not significantly different in pairs of strains originating from the same patient or from different patients, either from the patient’s airways or domestic environment with similar distributions in the interaction type (Figure 2). However, interactions affecting growth and motility were significantly more frequent for pairs of clinical strains and those comprising at least one clinical strain compared with pairs of environmental strains (Figure 2).

Under the study conditions, competition was significantly more frequent for pairs that had a *Pa* and/or an *Ax* strain, 38.5% (57/148) and 36.8% (35/95), respectively, compared with those comprising an *Sm* and/or an *Sa* strain, 22.9% (24/105) and 20% (5/25), respectively (*P*-value < 0.05; Table 2).

Interspecies interactions evaluated through 170 co-cultures of which 155 (91.2%) included an emerging pathogen, *Ax* or *Sm*, are presented in Table 2. They were observed in both intrapatient (30 pairs of strains out of 101 tested) and interpatient (24/69) conditions. *Pa* outcompeted its bacterial partner in the majority of the cases, except in 16 crossing experiments where growth, motility and/or pigmentation of 10 *Pa* strains were affected in co-culture with *Ax* (seven strains) or *Sm* (one strain; Table 2 and Supplementary Table 2), nine pairs of which were later included in the in-depth study. Interestingly, the three variants of *Ax* (*Ax* 198, *Ax* 199, and *Ax* 200) of identical MLST but distinct colony morphotypes co-cultured from a sputum specimen showed different competitive ability toward *Pa* strains (Supplementary Table 1). The first data on competition assays between *Ax* and *Sm* showed that *Ax* outcompeted *Sm* in all cases, either by decreasing *Sm* growth (*n* = 1, *Ax*/*Sm* pair of isolates from the environment of patients C and A, respectively) or motility (*n* = 4). Finally, out of eight pairwise comparisons between *Sa* and *Sm*, one interaction affecting *Sm* motility was observed (12.5%).

Intraspecific competitions were observed for *Pa* and *Ax* only in both intrapatient (nine pairs of strains out of 20 tested) and interpatient (4/13) conditions. For *Pa*, competition mostly affected bacterial growth (Table 2) and was mainly observed for pairs of clinical/environmental strains (*n* = 6 out of nine pairs) and of different genotypes (*n* = 5; Supplementary Table 1). In these cases, the *Pa* environmental strain decreased the growth of the clinical isolate. Unlike the observations made for *Pa*, all four modifications observed in the intra-*Ax* assays concerned clinical strains and bacterial motility (Table 2 and Supplementary Figure 3).

### In-Depth Study on 9 Selected Strains From Three Patients

#### Intermittent *Sm* (ST 5) Versus Sporadic *Pa* (ST 253) Colonizing Patient E

**FIGURE 3:**
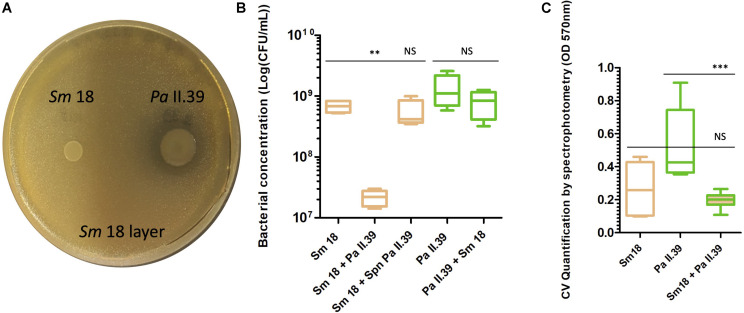
Interactions between intermittent *Stenotrophomonas maltophilia* (*Sm* 18, ST 5) and sporadic *Pseudomonas aeruginosa* (*Pa* II.39, ST 253) isolated about 1-year apart in Patient E. **(A)** Inhibition of *Sm* 18 growth by *Pa* II.39 during competition tests on bacterial layer after 48 h of co-culture. **(B)** Bacterial concentration after 48 h of culture in liquid medium of *Sm* 18 and *Pa* II.39 in monoculture, co-culture or *Sm* culture in presence of *Pa* supernatant. CFU, colony forming unit; Spn, supernatant. **(C)** Quantification of biofilm formed after 48 h of monoculture or co-culture. CV, crystal violet. The color indicates the species whose cells are numbered in **(B)** orange for *Sm* and green for *Pa*. The same color code is used in **(C)** to indicate the species whose biofilm formation has been measured; hatched box-plots with mixed colors indicate global bacterial quantification in biofilm experiments. NS, not significant; ^∗∗^, significant *P*-value < 0.01, ^∗∗∗^, significant *P*-value < 0.001.

The *Sm* 18 growth inhibition observed during the screening assay (mean inhibition zone diameter of 21.6 mm; Figure 3A) was confirmed by growth curve analysis showing a significant decrease in *Sm* growth in the presence of *Pa* II.39 (by a factor of 23.2 after 24 h, *P*-value < 0.001 and of 31.3 after 48 h, *P*-value < 0.01) while the growth of *Pa* remained unchanged in the presence of *Sm* (Figure 3B). *Pa* supernatant had no inhibitory effect on *Sm* suggesting that the competition between both species required a direct contact between the two bacteria (Figure 3B). Regarding biofilm formation, *Sm* 18 and *Pa* II.39 were moderate and strong biofilm producers, respectively. During co-culture, a significant decrease in dual-species biofilm production was observed (*P*-value < 0.001) in comparison with *Pa* biofilm (Figure 3C) despite the unchanged growth of *Pa* in planktonic culture.

#### Chronic *Sa* (ST 5) of Patient D Versus Sporadic *Pa* Originating From the Same Patient (ST 1062) and a Sporadic *Pa* From Another Patient (ST 253)

**FIGURE 4:**
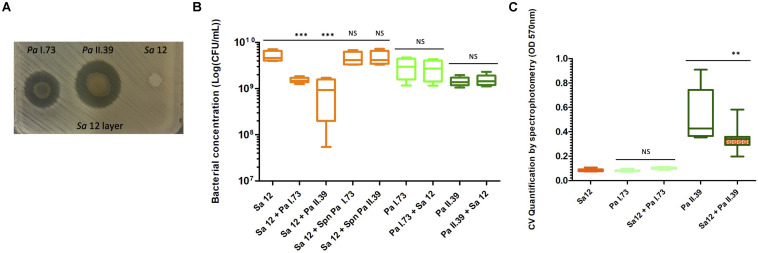
Interactions between chronic *Staphylococcus aureus* (*Sa* 12, ST 5) versus sporadic *Pseudomonas aeruginosa* originating from Patient D (*Pa* I.73, ST 1062, same sample than *Sa* 12) and a sporadic *P. aeruginosa* from Patient E (*Pa* II.39, ST 253). **(A)** Inhibition of *Sa* 18 growth by *Pa* I.73 and *Pa* II.39 during competition tests on bacterial layer after 48 h of co-culture. **(B)** Bacterial concentration after 48 h of culture in liquid medium of *Sa* 12, *Pa* I.73, and *Pa* II.39 in monoculture, *Sa*/*Pa* co-cultures or *Sa* culture in presence of *Pa* supernatant. CFU, colony forming unit; Spn, supernatant. **(C)** Quantification of biofilm formed after 48 h of monoculture or co-culture. CV, crystal violet. The color indicates the species and strains whose cells are numbered in **(B)** orange for *Sa*, light green for *Pa* I.73, and dark green for Pa II.39. The same color code is used in **(C)** to indicate the species and strains whose biofilm formation has been measured; hatched box-plots with mixed colors indicate global bacterial quantification in biofilm experiments. NS, not significant; ^∗∗^, significant *P*-value < 0.01, ***, significant *P*-value < 0.001.

In screening experiments, *Sa* 12 growth was inhibited by both *Pa* strains; however, the strain originating from the same patient (*Pa* I.73) had limited inhibition (inhibition zone diameter of 23.2 mm after 48 h) compared to *Pa* II.39 from another patient (31 mm; Figure 4A). This was confirmed by drawing a growth curve showing that, after both 24 and 48 h of liquid co-culture, the growth curves of *Pa*/*Sa* pairs of strains showed a significant decrease in *Sa* growth (by a factor of 3.3 and 4.7 after 24 h and by a factor of 3.4 and 5.7 after 48 h for both *Pa*/*Sa* pairs, respectively, *P*-value < 0.001) and no modification in *Pa* growth (Figure 4B). However, any *Pa* supernatants affected the growth of *Sa*, suggesting that direct contact mediated the outcompetition of *Sa* by *Pa* (Figure 4B). A growth inhibition of an adapted *Sa* strain, chronically colonizing the patient airways, was observed in both cases but was different according to origin, i.e., from the same patient or from a different patient, and/or genotype of the *Pa* strain although not significantly (*P*-value = 0.06). Finally, a significant decrease in dual-species biofilm formation was observed for strains originating from different patients alone compared with *Pa* II.39 biofilm formation, although growth of the *Pa* strain remained unchanged in planktonic culture (*P*-value < 0.01; Figure 4C).

#### Chronic *Ax* (ST 327) Versus Three Sporadic *Pa* of Different Genotypes (ST 27, ST 244 and ST 1092) and an Environmental *Pa* (ST 27) in Patient A

**FIGURE 5:**
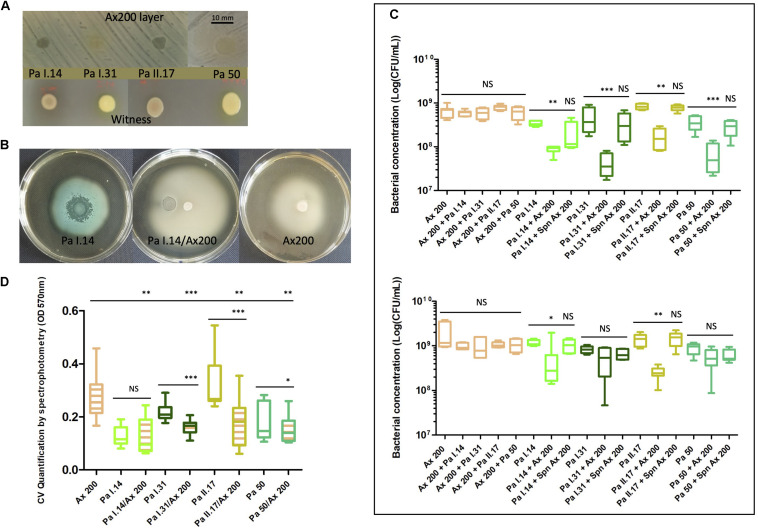
Interactions between chronic *Achromobacter xylosoxidans* (*Ax*; ST 327) versus three sporadic *Pseudomonas aeruginosa* (*Pa*) of different genotypes (ST 27, ST 244 and ST 1092) and an environmental *Pa* (ST 27) in Patient A. Strain isolation characteristics are presented in Supplementary Figure 2. **(A)** Inhibition of *Pa* I.14, *Pa* I.31, *Pa* II.17, and *Pa* 50 development on *Ax* 200 layer after 48 h of co-culture compared to corresponding monocultures. **(B)** Inhibition of *Pa* I.14 pigmentation and swimming motility during the co-culture with *Ax* 200 compared to monocultures. **(C)** Bacterial concentration after 24 h **(top)** and 48 h **(bottom)** of culture in liquid medium of *Ax* 200, *Pa* I.14, *Pa* I.31, *Pa* II.17, and *Pa* 50 in monocultures and *Ax*/*Pa* co-cultures or *Pa* culture in presence of *Ax* supernatant. CFU, colony forming unit; Spn, supernatant. **(D)** Quantification of biofilm formed after 48 h of monoculture or co-culture. CV: crystal violet. The color indicates the species and strains whose cells are numbered in **(C)** and **(D)** orange for *Ax*, light green for *Pa* I.14, and dark green for *Pa* I.31, light brown for *Pa* II.17, and turquoise for the environmental *Pa* 50. The same color code was used in C to indicate the species and strains whose biofilm formation has been measured; hatched box-plots with mixed colors indicate global bacterial quantification in biofilm experiments. NS, not significant; ^∗^, significant *P*-value < 0.05; ^∗∗^, significant *P*-value < 0.01; ^∗∗∗^, significant *P*-value < 0.001.

A timeline representation of selected strains in Patient A is shown in  Supplementary Figure 2. Strains were selected based on the results of screening assays, particularly on a marked inhibition of *Pa* growth, pigmentation, and motility in presence *Ax* 200 (Figures 5A,B). After co-culturing clinical strain *Ax* 200 (the most competitive *Ax* strain in screening assays) with each of the four selected *Pa* strains in liquid medium for 24 h, the growth of all *Pa* was significantly reduced (by a factor of 3.9–10.8, *P*-value < 0.01; Figure 5C) whereas no modification in *Ax* growth was observed. The *Ax* supernatant decreased *Pa* growth by a factor of 1.1–1.6 but this was not significant (Figure 5C). After 48h of co-culture, the growth of *Ax* 200 was not decreased by any of the four *Pa* strains whereas *Ax* 200 retained the ability to significantly inhibit the growth of *Pa* I.14 and of the co-isolated *Pa* strain (*Pa* II.17; *P*-value < 0.05). The effect of the *Ax* 200 supernatant remained unchanged (with no significant inhibitory effect on *Pa* growth decreased by a factor of 0.9–1.4; Figure 5C). We also explored growth of *Ax* and *Pa* on intra-clinical samples by co-culturing each of the three *Ax* variants, *Ax* 198, *Ax* 199, and *Ax* 200, with *Pa* II.17. *Pa* growth was slightly, but not significantly, reduced by *Ax* 198 (factor 2, *P*-value = 0.07) whereas it was significantly reduced by *Ax* 199 and *Ax* 200 (factors 3.9 and 5, respectively, *P*-value < 0.05; Supplementary Figure 4A). After 48 h of co-culturing, similar results were observed (data not shown). A distinct ability to form biofilm was noted for the seven strains studied in Patient A, the three *Ax* variants being either moderate biofilm producers (*Ax* 198) or strong biofilm producers (*Ax* 199 and *Ax* 200) and the four *Pa* strains being either low biofilm producers (*Pa* I.14), moderate biofilm producers (*Pa* I.31 and the environmental *Pa* 50) or strong biofilm producers (*Pa* II.17). Under all conditions of co-culture, a significant reduction in biofilm production was observed for *Pa* and/or *Ax* compared with each of the monocultures (*P*-value < 0.05; Figure 5D and Supplementary Figure 4B) unrelated to growth modifications observed for planktonic cultures except for *Pa* II.17 in co-culture with *Ax* 200 or *Ax* 199.

## Discussion

### Competitive Abilities of Emerging CF Pathogens

Concerning bacterial competition that may occur in the CF lung, most studies have always focused on *Pa* and deciphering the mechanisms of bacterial interactions with other opportunistic pathogens by studying the communication signals of its QS and secretion systems. As *Pa*–*Sa* co-isolation is common in CF, a majority of studies have focused on these opportunistic pathogens (Baldan et al., 2014; Filkins et al., 2015; Hotterbeekx et al., 2017; Limoli et al., 2017; Tognon et al., 2017) whereas fewer studies have addressed the question of interactions between *Pa* and other bacteria such as members of the *Burkholderia cepacia* complex (Al-Bakri et al., 2004; Schwab et al., 2014; Bragonzi et al., 2012; Smalley et al., 2015) and various members of the commensal microbiota of the CFRT (Shinzato and Saito, 1994; Sibley et al., 2008; Whiley et al., 2014; Gao et al., 2018). *Pa* has generally proved to outcompete other species, but more complex and reciprocal interactions between species have been further demonstrated with protection from killing by antimicrobial agents conferred by *Pa* to other species or modulation or potentiation of *Pa* metabolism and virulence by commensal members of the host microbiota like streptococci, *Gemella* or anaerobes (Duan et al., 2003; Hoffman et al., 2006; Michelsen et al., 2014; Whiley et al., 2015; Gao et al., 2018).

*Ax* and *Sm* are considered as emerging in CF (Lyczak et al., 2002; Lambiase et al., 2011; Parkins and Floto, 2015; Esposito et al., 2017). Although these bacteria may be co-isolated with *Pa* and/or *Sa* and have the ability to persistently colonize patients’ airways, so far no study has evaluated the competitive ability of members of the genus *Achromobacter* in CF and *Sm* competitive ability has rarely been studied in the context of CF (Pompilio et al., 2015; Magalhães et al., 2017; McDaniel et al., 2020).

#### Interactions Between *Sm* and *Pa*

In our study of 10 *Sm* and 15 *Pa* strains, we showed that *Pa*/*Sm* interactions affecting bacterial growth were the most frequent and that, in all cases, *Sm* growth was markedly inhibited by *Pa*, as previously observed for a couple of strains co-isolated from the lung of a chronically infected CF patient (Pompilio et al., 2015). However, several interactive traits were specifically observed in our study: (i) when motility modifications were observed, *Pa* motility increased systematically, unlike the observation made by Pompilio and colleagues (Pompilio et al., 2015); (ii) *Pa* virulence modulation by *Sm* previously reported is indirectly confirmed by global modifications to *Pa* pigment production. A couple of strains for which *Pa* pigment production decreased in our study suggested a potential decrease in *Pa* virulence caused by certain *Sm* strains which has never previously been described (Pompilio et al., 2015), and (iii) a significant decrease in *Pa* biofilm formation unlike previous studies showing either a significant increase in biofilm biomass between a CF *Sm* strain and a *Pa* reference strain (Magalhães et al., 2017) or no difference in total biofilm biomass after 6 days of mixed cultures of CF clinical strains (Pompilio et al., 2015). Interactions not previously reported require further investigations to specify whether they could be strain-dependent, according to strain’s origin, genotype or context of isolation, and/or mediated by specific mechanisms.

#### *Ax* Competitive Ability

This study showed interspecies interactions mainly in *Ax*/*Pa* tests and, to a lesser extent, in the *Ax*/*Sm* assays. We reported the first data on competition between *Ax* and *Sm* showing that *Ax* outcompeted *Sm* in all cases. A more complex interplay was noted between *Ax* and *Pa*, with *Pa*’s growth, motility and/or pigment production being either increased or decreased. In the current context wherein *Pa* is recognized as a highly competitive microorganism and, in the absence of any data on *Ax*/*Pa* competition, this prompted us to investigate pairs of strains in which *Ax* outcompetes *Pa* more thoroughly. This revealed additional competition in terms of biofilm formation with the biomass of *Ax*/*Pa* biofilms being significantly reduced compared with monocultures, as previously observed for *Pa*/*Sa* pairs (Magalhães et al., 2017; this study), and for *Pa* and the unusual CF pathogen *Inquilinus limosus* (Magalhães et al., 2017). The ability of *Ax* to form biofilm has been previously demonstrated (Trancassini et al., 2014; Filipic et al., 2017) but interactive modifications have never been described before. In our study, we showed that biofilm formation was disturbed during the interaction between *Ax* and *Pa* and, unlike the observations made earlier for *Sm* (Pompilio et al., 2015; Magalhães et al., 2017) and *Burkholderia cenocepacia* (Bragonzi et al., 2012), neither partner cooperated and both reduced their biofilm productions. This observation, associated with the growth modifications in *Ax*/*Pa* co-cultures, is highly suggestive of reciprocal interactions between *Ax* and *Pa* as previously described between *Pa* and other species like *Sa*, *Sm*, and *B. cepacia* complex (Bragonzi et al., 2012; Pompilio et al., 2015; Limoli et al., 2017; Magalhães et al., 2017; Tognon et al., 2017).

We therefore underline the need for additional studies on emerging CF pathogens to better delineate their importance in the complex interactions taking place in CF airways.

### Multiple Interactions Suggestive of QS-Regulated Competition

Multiple types of interactions were observed for a quarter of the pairs of strains for which competition was noted in our study. The induction of *Pa* pigment production, in particular, was systematically associated with another effect on growth or bacterial motility. This emphasizes that various competition strategies could be established simultaneously between microorganisms (Köhlerx et al., 2009) and suggests a regulation by a common QS mechanism (Smalley et al., 2015). Competition systems have been largely described for *Pa* (Tashiro et al., 2013; Schwab et al., 2014; Filkins et al., 2015; Smalley et al., 2015; Bernier et al., 2016). For *Sa*, the major QS system, the accessory gene regulator (*agr*) system, has also been widely studied (Le and Otto, 2015). By contrast, mechanisms supporting *Pa*/*Sm* interactions have yet to be more deeply deciphered. At present, studies have shown that the *Sm* antimicrobial activity exhibited by *Pa* is not mediated by components of the *Pa* supernatant but established in a contact-dependent manner (Pompilio et al., 2015; this study). Others underline a role for signal molecules of the diffusible signal factor (DSF) family, *cis*-2-unsaturated fatty acids, produced by *Sm* on *Pa* virulence, persistence, biofilm formation, and stress tolerance (Ryan et al., 2008; Twomey et al., 2012), as well as a pro-killing effect of *Sm* on *Pa* involving the type IVA secretion system recently described in *Sm* (Nas et al., 2019). In our study, *Pa* culture supernatants had no effect on *Sa* growth and this result must be further investigated as it contrasts with previous studies showing that *Pa* exoproducts determine antimicrobial activity against *Sa* (Radlinski et al., 2017). Finally, the mechanisms of *Achromobacter* QS are not yet elucidated even though cyclic-di-GMP has been suspected of acting in QS signaling associated with biofilm formation as previously demonstrated for *Pa* (Valentini and Filloux, 2016; Nielsen et al., 2019). This also warrants further investigation.

### Complex Interactions Revealed Through the Study of a Large Panel of Documented Isolates

Present studies, apart from three evaluating intraspecific *Pa* interactions (Ghoul et al., 2015; Chatterjee et al., 2017; Bara et al., 2018) and one multispecies study (Bernier et al., 2016), including more than 50 strains, mostly include limited numbers of strains. Similarly, most published studies include reference strains. Taking *Pa* as an example, strains PAO1 and PA14 are regularly included in studies on CF bacterial interactions as the sole *Pa* strains (Beaume et al., 2015; Smalley et al., 2015; Bernier et al., 2016; Magalhães et al., 2017; Tognon et al., 2017; Anand et al., 2018). However, these two strains, originating from a human wound and a burns patient, respectively (Klockgether et al., 2010; Mathee, 2018), may not reflect the true behavior of CF clinical strains. When clinical CF strains are studied, with a few exceptions, there is usually no information given on the colonization history or profile of patients and these are major factors in the competitive traits of bacterial isolates. Indeed, isolates from early and later stages of infection have previously been shown to have distinct competitiveness (Michelsen et al., 2014; Ghoul et al., 2015; Morgan et al., 2020) or differences in gene expression that may influence the strains’ competitive ability (Nielsen et al., 2017). Finally, little information on the genotypes of clinical strains is usually available in these studies.

Enlarging the panel of clinically documented strains tested in competition assays in our study showed divergent competitive ability between species depending on the pairs of strains under consideration – from indifference to competition – affecting the three characteristics under evaluation and among strains of a same species. These results completed scarce available observations as follows: (i) intraspecific interactions previously demonstrated for *Pa* alone were observed in our study for the two species *Pa* and *Ax* (Chatterjee et al., 2017; Bara et al., 2018; Oluyombo et al., 2019); (ii) considering the strain’s clinical or environmental origin, a significant higher rate of interactions was observed among clinical strains and between clinical and environmental strains, compared with pairs of environmental strains with a free lifestyle. Only two previous studies included *Pa* clinical CF and free-living environmental strains (Chatterjee et al., 2017; Bara et al., 2018), one of which included strains from CF homes (Bara et al., 2018). These studies showed that CF and environmental isolates did not differ significantly in their competitive ability (Bara et al., 2018) and that similar rates of antagonistic interactions were observed between environmental and clinical strains, and among environmental strains, although some environmental strains inhibited clinical strains (Chatterjee et al., 2017); and finally (iii), we found divergent competitive ability depending on the context of isolation (from the same patient, the same sample or from different patients) and among distinct but clonally related morphotypes isolated from one sample, resulting from diversification within adapted bacterial populations (Dupont et al., 2015).

### *Ax* Competitiveness and Colonization Pattern of CF Airways

Major bacterial pathophysiological traits represented by growth, motility, biofilm formation, and *Pa* pigment production were shown to be affected by bacterial competition for a third of the pairs of strains in our study. Interpreting the consequences of these interactions remains a challenge (Hibbing et al., 2010) as species may be co-localized or not in the CF lung and as, for most interactions, dual interpretations, either beneficial or deleterious for a bacterium, can be drawn. The example of flagellar motility modifications is iconic. Flagellar motility is indeed a key parameter of bacterial adhesion and biofilm formation and its inhibition was shown important for stabilizing cell aggregates in several species including *Pa* (Guttenplan and Kearns, 2013). Recently, the downregulation of flagellar motility has also been confirmed as decisive for biofilm formation in *Ax* (Nielsen et al., 2019) although a previous study on 69 clinical isolates did not find any correlation between swimming phenotype and biofilm formation (Filipic et al., 2017). However, the interspecific, intraspecific, and intraclonal increase in swimming motility observed in our study represents another opportunity to persist within the CF airways, in the same way as the “exploratory motility” recently studied in *Pa* (Limoli et al., 2019).

The involvement of such highly complex interactions in the pulmonary colonization of CF patients and its dynamics remains to be elucidated. Some studies highlight the importance of the colonization sequence in driving cooperation and competition between CF *Pa* strains and oral commensal streptococci (Whiley et al., 2015) and also among *Pa* strains (Qin et al., 2012). Intraspecific interactions may explain certain observations that implanting a second strain of *Pa* in a patient who is already colonized by *Pa* has little chance of success (Qin et al., 2012). Although no specific studies have been made on *Ax*, we previously demonstrated that patients chronically colonized by *Ax* harbored highly phenotypically and genotypically diversified *Ax* populations but that all the variants observed were clonally related (Dupont et al., 2015; Dupont et al., 2016). As previously observed, no unrelated *Ax* strains were able to become implanted in these patients (Ridderberg et al., 2011; Amoureux et al., 2013). In our study, for patient A chronically colonized by *Ax* and sporadically by *Pa* and for whom the highest number of strains were studied, we found that complex, reciprocal competition between these two species occurred. However, our major observation was the ability for clinical strains of *Ax* to outcompete clinical and environmental *Pa*. These observations, highly atypical in the overall literature dealing with bacterial competition in CF, questioned the importance of bacterial competitiveness in the colonization pattern of CF airways when linked to the patient’s colonization history. Finally, in Patient A, we also observed distinct competitive ability between *Ax* strains of identical MLST genotypes but distinct cultural morphotypes generated through adaptation during persistent colonization. Intraspecies interactions between these adaptive *Ax* variants found in chronic colonization may confer a selective advantage to some variants among the *Ax* community that may contribute to the species persistence in the patient’s airways (Limoli et al., 2019).

## Conclusion and Outlook

Despite the limitations of not having explored further mechanisms of the observed interactions and the fact that this was an in-depth study conducted only on selected strains, the results of our study on 39 CF-related strains with clinical and genotypic documentation provide new perspectives on the complex interactions that may take place among the CF airway community. This is the first study describing the competition capacity of *Ax* strains, adding new findings to the scarce data on *Sm* strains and highlighting the importance of bacterial competitiveness in the colonization pattern of CF airways. A broader exploration of the bacterial interactions between *Ax* and *Pa* strains, by diversifying sources of isolates (clinical non-CF, hospital environment, natural environments) is required to search for specific competition that might be established in the lungs of CF patients. It would also be interesting to test *Achromobacter* species other than *Ax* which are prevalent in CF patients (Spilker et al., 2013). We recommend screening as a valuable first-stage strategy to detect the most competitive strains before selecting strains for further testing. Besides additional *in vitro* investigations on pigment production quantification, the decrease in *Pa* virulence by some *Ax* strains must be specified using *in vivo* models such as *Caenorhabditis elegans* and/or zebrafish embryos as previously used for CF pathogens (Clatworthy et al., 2009; Dupont et al., 2017). Finally, the role of QS systems also remains to be explored to understand the mechanisms underlying *Pa* inhibition by *Ax*.

## Data Availability Statement

The datasets presented in this study (the previously undescribed *Stenotrophomonas maltophilia* ST 486) can be found in online repositories. The names of the repository/repositories and accession number(s) can be found at: https://pubmlst.org/bigsdb?page=info&db=pubmlst_smaltophilia_isolates&id=654.

## Ethics Statement

The studies involving human participants were reviewed and approved by the Institutional Review Board at Nîmes University Hospital (IRB no. 19.02.01 for the study of clinical isolates and collection of patients’ microbiological results and IRB no. 15/07.05 for the study of isolates from the domestic environment and the sampling at the patients’ homes). Written informed consent from the participants’ legal guardian/next of kin was not required to participate in this study in accordance with the national legislation and the institutional requirements.

## Author Contributions

CD and HM conceived and designed the study. HM designed the method and collected the clinical isolates. CD, RC, and EJ-B designed the method, collected the environmental isolates, and critically revised the manuscript. RC collected the clinical data. QM, CD, and HM collected the microbiological data and analyzed and interpreted the data. QM and CD performed the microbial analyses. QM and HM drafted the manuscript. All authors read and approved the final manuscript.

## Conflict of Interest

The authors declare that the research was conducted in the absence of any commercial or financial relationships that could be construed as a potential conflict of interest.
