# Supplementary material for: High Occurrence of Bacterial Competition Among Clinically Documented Opportunistic Pathogens Including Achromobacter xylosoxidans in Cystic Fibrosis
Source: Front Microbiol. 2020 Sep 10;11:558160. doi: 10.3389/fmicb.2020.558160 (PMC7513574; doi:10.3389/fmicb.2020.558160)
Supplement: Supplementary file 1 [file Presentation_1.pptx]

## Slide 1
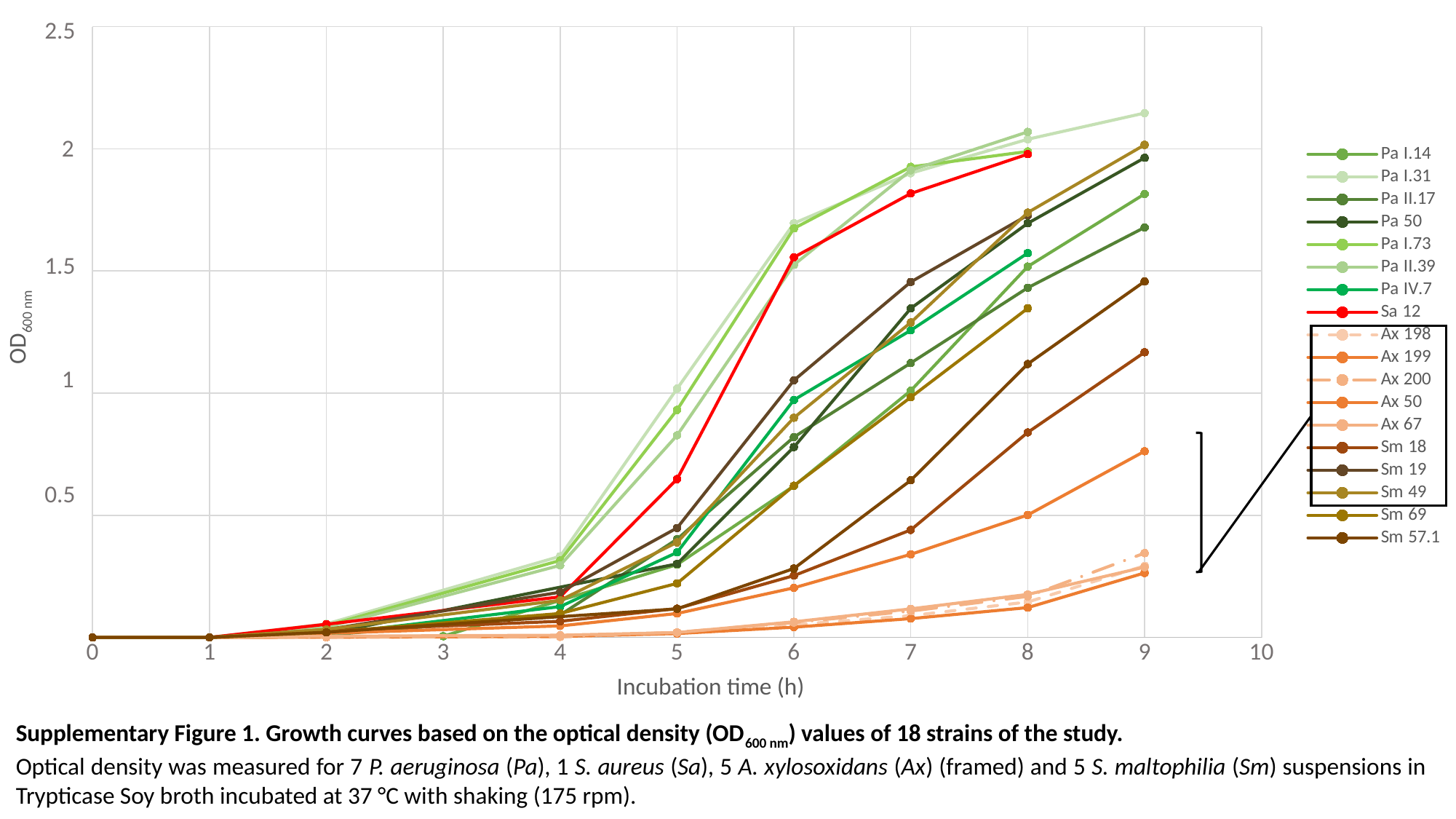

2.5
2
1.5
1
0.5
### Chart
| Category | Pa I.14 | Pa I.31 | Pa II.17 | Pa 50 | Pa I.73 | Pa II.39 | Pa IV.7 | Sa 12 | Ax 198 | Ax 199 | Ax 200 | Ax 50 | Ax 67 | Sm 18 | Sm 19 | Sm 49 | Sm 69 | Sm 57.1 |
|---|---|---|---|---|---|---|---|---|---|---|---|---|---|---|---|---|---|---|
| 0 | 0.0 | 0.0 | 0.0 | 0.0 | 0.0 | 0.0 | 0.0 | 0.0 | 0.0 | 0.0 | 0.0 | 0.0 | 0.0 | 0.0 | 0.0 | 0.0 | 0.0 | 0.0 |
| 1 | 0.0 | 0.0 | 0.0 | 0.0 | 0.0 | 0.0 | 0.0 | 0.0 | 0.0 | 0.0 | 0.0 | 0.0 | 0.0 | 0.0 | 0.0 | 0.0 | 0.0 | 0.0 |
| 2 | 0.003 | 0.055 | 0.009 | 0.015 | 0.048 | 0.041 | 0.012 | 0.054 | 0.001 | 0.001 | 0.002 | 0.017 | 0.005 | 0.027 | 0.032 | 0.033 | 0.021 | 0.021 |
| 3 | 0.005 | None | None | None | None | None | None | None | None | None | None | None | None | None | None | None | None | None |
| 4 | None | 0.332 | 0.092 | None | 0.315 | 0.295 | 0.126 | 0.166 | 0.002 | 0.004 | 0.003 | 0.047 | 0.009 | 0.066 | 0.185 | 0.152 | 0.098 | None |
| 5 | 0.298 | 1.018 | 0.402 | 0.301 | 0.931 | 0.827 | 0.348 | 0.648 | 0.017 | 0.016 | 0.021 | 0.098 | 0.02 | 0.118 | 0.448 | 0.389 | 0.221 | 0.117 |
| 6 | 0.62 | 1.695 | 0.82 | 0.779 | 1.674 | 1.525 | 0.972 | 1.556 | 0.052 | 0.042 | 0.061 | 0.203 | 0.064 | 0.253 | 1.052 | 0.899 | 0.621 | 0.282 |
| 7 | 1.01 | 1.9 | 1.123 | 1.347 | 1.925999999999999 | 1.911999999999999 | 1.257 | 1.817 | 0.089 | 0.077 | 0.108 | 0.34 | 0.117 | 0.44 | 1.455 | 1.289 | 0.983 | 0.643 |
| 8 | 1.518 | 2.039 | 1.431 | 1.695 | 1.989 | 2.069 | 1.573 | 1.978 | 0.145 | 0.122 | 0.168 | 0.501 | 0.176 | 0.839 | 1.728 | 1.739 | 1.347 | 1.119 |
| 9 | 1.815 | 2.146 | 1.678 | 1.963 | None | None | None | None | 0.293 | 0.264 | 0.345 | 0.762 | 0.288 | 1.167 | None | 2.016 | None | 1.457 |
| 10 | None | None | None | None | None | None | None | None | None | None | None | None | None | None | None | None | None | None |OD600 nm
Incubation time (h)
Supplementary Figure 1. Growth curves based on the optical density (OD600 nm) values of 18 strains of the study.
Optical density was measured for 7 P. aeruginosa (Pa), 1 S. aureus (Sa), 5 A. xylosoxidans (Ax) (framed) and 5 S. maltophilia (Sm) suspensions in Trypticase Soy broth incubated at 37 °C with shaking (175 rpm).

## Slide 2
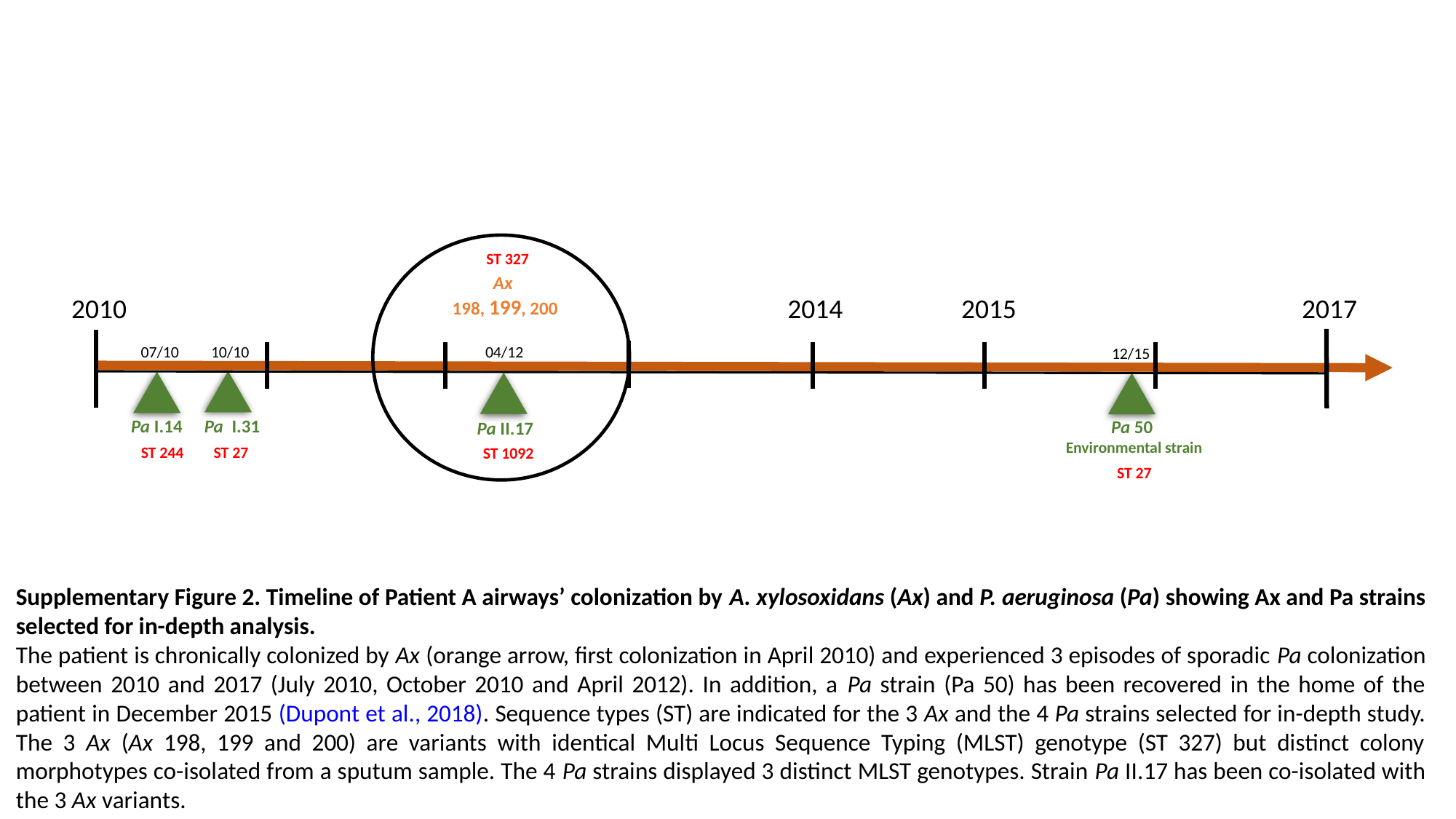

2010
2014
2017
10/10
04/12
12/15
Pa I.14
Pa I.31
Pa 50
Environmental strain
Pa II.17
Ax
198, 199, 200
07/10
2015
ST 327
ST 244
ST 27
ST 1092
ST 27
Supplementary Figure 2. Timeline of Patient A airways’ colonization by A. xylosoxidans (Ax) and P. aeruginosa (Pa) showing Ax and Pa strains selected for in-depth analysis.
The patient is chronically colonized by Ax (orange arrow, first colonization in April 2010) and experienced 3 episodes of sporadic Pa colonization between 2010 and 2017 (July 2010, October 2010 and April 2012). In addition, a Pa strain (Pa 50) has been recovered in the home of the patient in December 2015 (Dupont et al., 2018). Sequence types (ST) are indicated for the 3 Ax and the 4 Pa strains selected for in-depth study. The 3 Ax (Ax 198, 199 and 200) are variants with identical Multi Locus Sequence Typing (MLST) genotype (ST 327) but distinct colony morphotypes co-isolated from a sputum sample. The 4 Pa strains displayed 3 distinct MLST genotypes. Strain Pa II.17 has been co-isolated with the 3 Ax variants.

## Slide 3
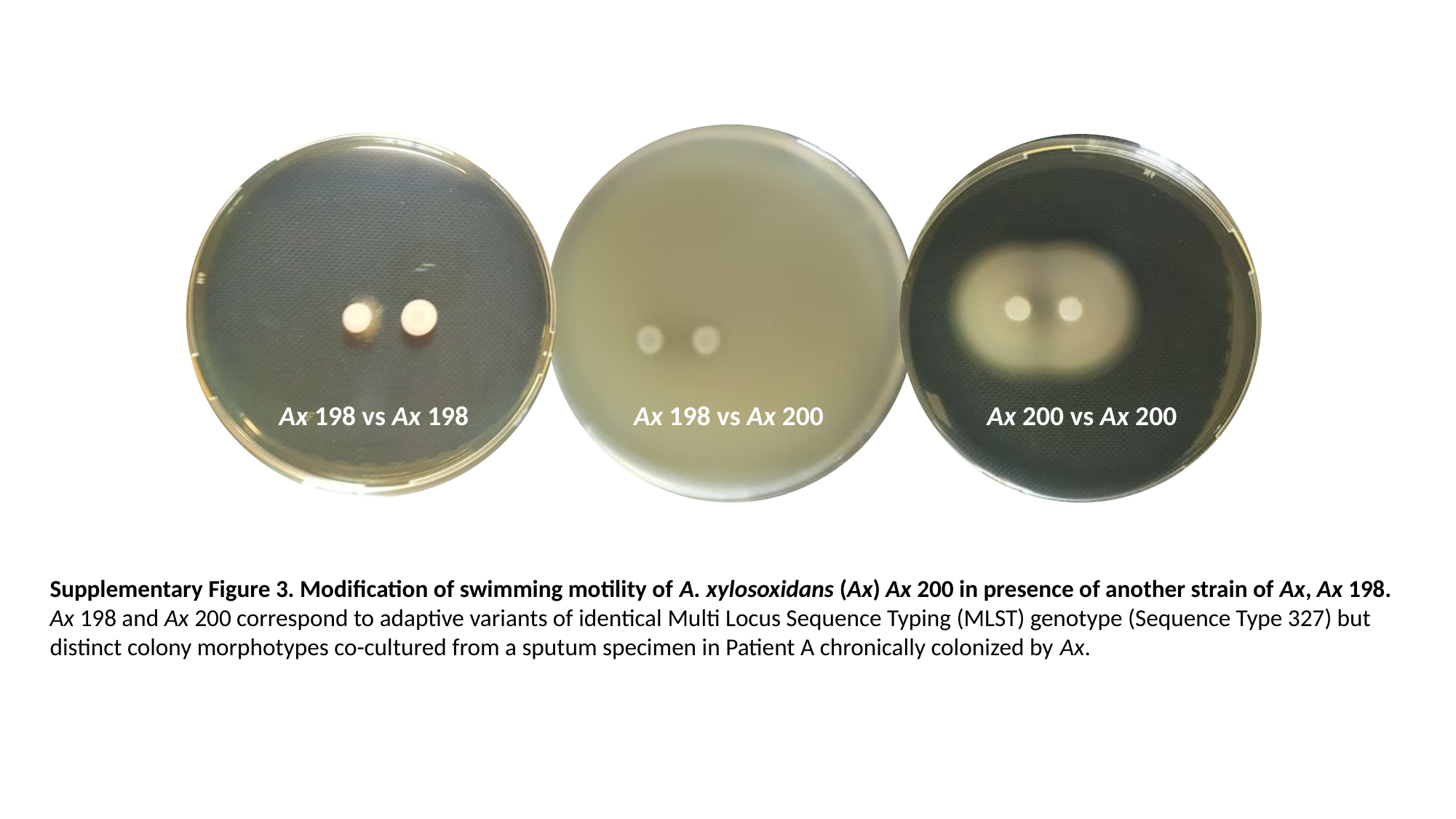

Ax 198 vs Ax 198
Ax 198 vs Ax 200
Ax 200 vs Ax 200
Supplementary Figure 3. Modification of swimming motility of A. xylosoxidans (Ax) Ax 200 in presence of another strain of Ax, Ax 198.
Ax 198 and Ax 200 correspond to adaptive variants of identical Multi Locus Sequence Typing (MLST) genotype (Sequence Type 327) but distinct colony morphotypes co-cultured from a sputum specimen in Patient A chronically colonized by Ax.

## Slide 4
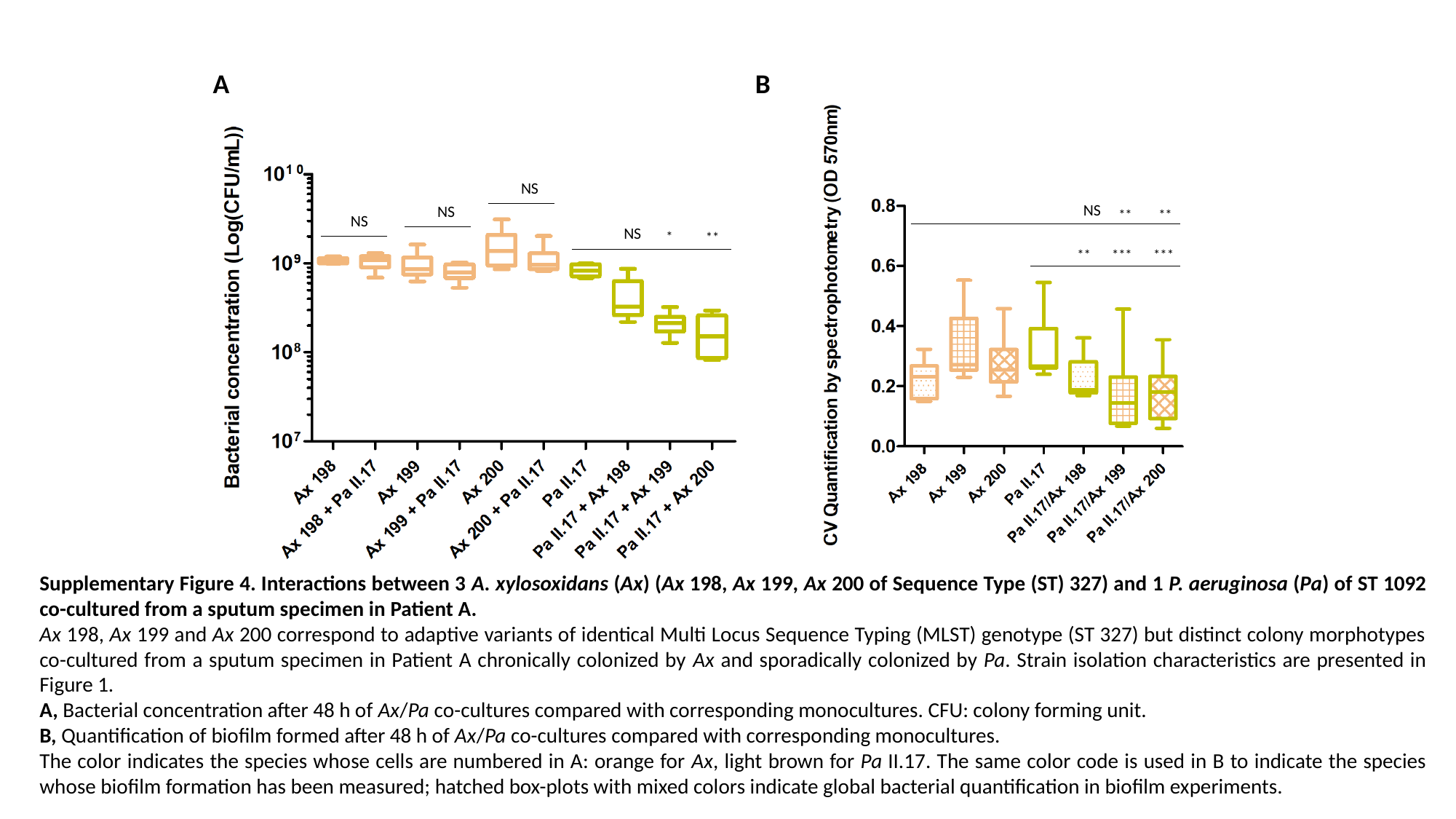

B
NS
**
**
**
***
***
A
NS
NS
NS
NS
*
**
Supplementary Figure 4. Interactions between 3 A. xylosoxidans (Ax) (Ax 198, Ax 199, Ax 200 of Sequence Type (ST) 327) and 1 P. aeruginosa (Pa) of ST 1092 co-cultured from a sputum specimen in Patient A.
Ax 198, Ax 199 and Ax 200 correspond to adaptive variants of identical Multi Locus Sequence Typing (MLST) genotype (ST 327) but distinct colony morphotypes co-cultured from a sputum specimen in Patient A chronically colonized by Ax and sporadically colonized by Pa. Strain isolation characteristics are presented in Figure 1.
A, Bacterial concentration after 48 h of Ax/Pa co-cultures compared with corresponding monocultures. CFU: colony forming unit.
B, Quantification of biofilm formed after 48 h of Ax/Pa co-cultures compared with corresponding monocultures.
The color indicates the species whose cells are numbered in A: orange for Ax, light brown for Pa II.17. The same color code is used in B to indicate the species whose biofilm formation has been measured; hatched box-plots with mixed colors indicate global bacterial quantification in biofilm experiments.
